# Acute Intermittent Porphyria in the North of China: The Acute Attack Effect on Quality of Life and Psychological Condition

**DOI:** 10.1155/2018/3216802

**Published:** 2018-05-15

**Authors:** Jing Yang, Tienan Zhu, Yongqiang Zhao, Xuezhong Yu, Huadong Zhu, Yinan Jiang, Xiaoqing Li

**Affiliations:** ^1^Emergency Department, Peking Union Medical College Hospital, Chinese Academy of Medical Sciences and Peking Union Medical College, China; ^2^Department of Hematology, Peking Union Medical College Hospital, Chinese Academy of Medical Sciences and Peking Union Medical College, China; ^3^Department of Psychology, Peking Union Medical College Hospital, Chinese Academy of Medical Sciences and Peking Union Medical College, China; ^4^Department of Gastroenterology, Peking Union Medical College Hospital, Chinese Academy of Medical Sciences and Peking Union Medical College, China

## Abstract

**Background:**

Acute intermittent porphyria (AIP) is an autosomal recessive disorder with intermittent attacks. Patients with AIP are susceptible to impaired quality of life and psychological distress.

**Objectives:**

To document the clinical features of AIP and its impact on SF-36 and IES scores of AIP patients in China and to explore the variables associated with SF-36 and IES scores.

**Methods:**

A single investigator collated data related to treatments and outcomes in 27 patients with AIP of PUMCH. A cross-sectional questionnaire survey including the SF-36, the IES-R, and demographic questions was conducted in the north of China. Differences in the QoL scale/summary scores and proportions in the QoL dimensions between patients and the general population were analyzed. Independent effects of chronic conditions and demographic variables on the SF-36 and IES-R were analyzed.

**Results:**

AIP patients had considerably lower SF-36 scores than the general population (the PF score and MH were lower than normal, *P* < 0.05). Working had higher RP than staying at home (*P* = 0.02); “without acute attack” had higher PF and BP scores and PCS composite score (*P* = 0.001). The mean IES-R score of AIP was higher than normal (36.7 ± 11.8 points, *P* < 0.001), “without acute attack” had lower intrusion score than “with acute attack” (*P* = 0.03).

**Conclusion:**

AIP patients in China had impaired quality of life, especially in terms of physical health. The acute attacks coursed the posttraumatic stress disorder-related symptoms.

## 1. Introduction

Acute intermittent porphyria (AIP, OMIM 176000) is a rare autosomal dominant disorder, caused by a partial deficiency of porphobilinogen deaminase (PBGD), with the alternative name hydroxymethylbilane synthase (HMBS), the third enzyme in the heme biosynthetic pathway [1]. The presentation of AIP is highly variable and the symptoms are nonspecific. The typical manifestations of AIP are intermittent attacks of abdominal pain, constipation, or confusion and convulsion occurs in some individuals [2]. In mainland of China, hematin is not available to treat acute porphyria. Fortunately, with high carbohydrate intake and the avoidance of harmful drugs, our patients almost completely recovered from their acute attacks, although their recovery required several weeks or months. The clinical features of AIP have been described in various case reports and small series, while larger studies have focused on particular aspects of the disease, such as genetics [2–5]. In addition, rare study has addressed the psychosocial consequences of AIP despite the apparent severity of symptoms and substantial changes to lifestyle necessary to avoid episodes of acute attack [6]. Bullinger et al. defined health-related quality of life (HRQoL) as “the impact of perceived health on an individual's ability to live a fulfilling life,” and it is now an important health outcome measure [7]. The MOS 36-item Short-Form Health Survey (SF-36) is a popular HRQoL measure that has been translated and validated for Chinese adults in Hong Kong (HK) [8, 9]. The effects of the SF-36 scores on consultation rates were stronger than those of sociodemographic and morbidity factors [8, 10]. Impact of Event Scale-Revised (IES-R) is a questionnaire that assesses posttraumatic stress disorder-related symptoms (PTSS) for the previous one-week period [11]. Here we reported findings of 27 patients with AIP in China, which were designed to record clinical features, methods of treatment impact on quality of life (QoL), and psychological condition of these AIP patients. To our knowledge, there were no such reports in Chinese population.

## 2. Compliance with Ethics Guidelines

The study design was approved by the Ethics Committee of the Institutional Review Board in Peking Union Medical College Hospital (PUMCH).

## 3. Patients and Methods

### 3.1. Patients

From January 2014 to December 2016, a total of 60 patients were diagnosed with acute intermittent porphyria in the emergency center and the gastroenterology department of PUMCH. The following diagnostic criteria were used: (1) acute attack symptoms and (2) being positive for urine porphobilinogen (PBG). Among the 60 AIP patients, 30 agreed to have genetic testing for PBGD gene mutations, 29 of 30 have proven the PBGD gene mutation, 10 of them have been reported [2], and 19 of them will be described in our other paper.

### 3.2. Procedures

Physicians determined eligible participants, informed them briefly about the study by phone or WeChat, and requested participation in the study. They got the answer link and a detailed explanation about the study via WeChat, with the link including self-report questionnaires. The questionnaires and medical information sheets were assigned an identification number; we did not make a correspondence list to ensure that participants remained anonymous. We contacted 28 of 29 who have proven PBGD gene mutation, and 27 of 28 patients finished the questionnaire.

### 3.3. Quality of Life Indices

Health-related quality of life (HRQoL) was measured using Chinese version 1.0 of the SF-36 Health Survey [13] developed in 1997. The SF-36 [14, 15] is made up of 36 questions that correspond to eight dimensions: physical functioning (PF), role physical (RP), bodily pain (BP), general health (GH), vitality (VT), social functioning (SF), role emotional (RE), and mental health (MH). All dimensions can be further classified into the physical domain (PF, RP, BP, and GH) and the mental domain (VT, SF, RE, and MH). The scores for each category scale from 0 to 100, in which 100 represents the best health status, while 0 represents the worst health status. The SF-36 scores can also be expressed as two summary measures, the physical component summary (PCS) and the mental component summary (MCS), which provides a measure of the overall effect of physical and mental impairment, with higher scores indicating better QoL [16].

### 3.4. Impact of Event Scale-Revised (IES-R)

IES-R is a 22-item self-report questionnaire that assesses posttraumatic stress disorder-related symptoms (PTSS) for the previous one-week period [11]. Symptoms are rated on a 5-point Likert scale (0–4), with higher scores indicating a high degree of PTSS. The IES-R consists of three components: intrusion, avoidance, and hyperarousal. We used the Chinese version of the IES-R [17] which demonstrated satisfactory validity and test-retest reliability. There is no specific cut-off score of IES-R. We classified participants to low PTSS (IES-R score < 26) or high PTSS (IES-R score ≥ 26) for analysis [18–20]; Cronbach's *α* was 0.95.

### 3.5. Genetic Testing

The molecular genetic testing of PBGD gene was performed by direct sequencing. All 14 exons of the PBGD gene and a minimum of 20 base pairs of flanking intronic DNA for each exon were amplified by polymerase chain reaction (PCR) (Tiangen Biotech, Beijing, China) and subsequently sequenced using the BigDye Terminator Cycle Sequencing Kit version 3.1 (ABI Biosystems) on ABI PRISM 3730 Sequence Analyzer according to the manufacturer's instructions.

### 3.6. Statistics

All statistical analyses were performed using SPSS software (IBM SPSS for Windows, version 16.0, SPSS, Inc., Chicago, IL) for descriptive analysis. Chi-square tests were employed for categorical variables, while parametric Student's *t*-tests or Mann–Whitney *U* tests were used for continuous counterparts, as appropriate. For all comparisons, a *P* value < 0.05 was considered to represent a significant difference. All statistical tests were 2-sided. The scores between categorical groups were compared using a *t*-test or one-way ANOVA as appropriate.

## 4. Results

### 4.1. Patients

The study cohort contained 27 patients (median age: 29 years, range: 21–47 years). The 27 patients were derived from 25 families.

### 4.2. Age at Onset and the Frequency of Acute Attacks

Age at onset and the frequency of acute attacks of AIP patients were summarized in Table 1. The median age at onset of symptoms was 26 years (range: 18–46 years), and the duration of history was 3.76 years (range: 1–8 years). 17/27 had acute attacks in the last year. The median frequency of acute attacks in the last year was 1.70 times (0–10 times), and the duration of every attack was 11.4 days (3–20 days).

### 4.3. Symptoms

The symptoms of AIP were summarized in descending order of frequency in Table 2. Other manifestations were shown such as dysphagia (*n* = 1). 9/27 had complaints in intermission, including fatigue, pain, or numbness.

### 4.4. Working Status

12/27 patients stayed at home, 4/27 worked at home, and 13/27 were employed. 22/27 thought they could work as usual.

### 4.5. Treatment

9/27 patients were treated with induced menopause lasting 1-2 years and 4/27 received the treatment of hematin (obtained in Hong Kong or Taiwan for once or twice).

### 4.6. Quality of Life

Scores for each subscale were PF, 85.74 ± 11.67 (91.83 with norm-based scoring [9], *P* = 0.01), RP, 64.81 ± 57.74 (82.43 [9], *P* = 0.13), BP, 77.96 ± 22.81 (83.98 [9], *P* = 0.18), GH, 51.67 ± 25.84 (55.98 [9], *P* = 0.39), VT, 57.96 ± 18.96 (60.27 [9], *P* = 0.53), SF, 85.65 ± 23.44 (91.19 [9], *P* = 0.23), RE, 69.13 ± 54.64 (71.67 [9], *P* = 0.81), MH, 65.19 ± 19.15 (72.79 [9], *P* = 0.049), PCS, 45.50 ± 10.39, and MCS, 47.76 ± 12.14.

Without acute attack with 2 significant subscales (PF and BP, *P* = 0.04) and 1 subscale trending toward significance than with acute attack group (GH with *P* = 0.05) and a lower PCS composite score (*P* = 0.001), one variable was associated with better QOL in the RP, working status (score of 35.42 for stay at home versus 88.33 for working; *P* = 0.02), as shown in Tables 3 and 4.

College education, marital status, and symptom severity were not significantly different across the composite or any of the subscales (Table 3).

### 4.7. Posttraumatic Stress Disorder

Posttraumatic stress disorder (PTSD) was measured by the IES-R questionnaire. The mean IES-R score was 36.7 ± 11.8 points (26 with norm-based scoring [21, 22], *P* < 0.001), and the average subscores were as follows: intrusion, 17.93 ± 5.78; avoidance, 18.48 ± 6.51; and hyperarousal, 13.26 ± 5.10.

We analyzed the clinical characteristics with IES-R score (Table 5). Only one variable was associated with better IES-R in 1 subscale (intrusion score: 14.44 with acute attack versus 19.53 without acute attack, *P* = 0.03), as shown in Table 5; neither were related to IES-R scores. College education, marital status, work status, and symptom severity were not significantly different across the composite or any of the subscales.

## 5. Discussion

The clinical manifestations of acute porphyria are diverse and occur as intermittent attacks that may be life-threatening [5, 23]. Although there have been considerable advances in the molecular genetics, pathophysiology, and management of patients with AIP, little was known on the psychosocial consequences of their diagnosis [6, 24]. In the mainland of China, hematin is not available to treat AIP and acute attacks are likely to result in hospitalization to intake high carbohydrate [2] for weeks. And the patients must be aware of precipitating events and medications. These factors suggest that psychosocial aspects are important variables in understanding the AIP patient experience. This study was focusing on self-rated psychosocial aspects and quality of life issues in AIP.

Demographic characteristics of this study sample showed similar trends found in our previous paper [2], in which 10 of 27 were described. Gastrointestinal symptoms were the most frequently reported complaints (abdominal pain and constipation) and neurological manifestations symptoms were more frequent than described in medical recording (Table 2, 13/27, 11/27 versus 10/36 [2], 5/36 [2]). Maybe some complaints would be ignored by Chinese clinicians.

It had been described that the perceived effects of 81 acute porphyria patients on quality of life and patient experience, depression, and particularly anxiety were more common than in the general population [24] (medical outcomes study general health questionnaire and EuroQol questionnaire). Our study used the SF-36 questionnaire and IES-R to assess the quality-of-life parameters and the prevalence of significant PTSD-related symptoms of the AIP patients.

The SF-36 has eight scales measuring eight domains of HRQoL; each scale consists of 2 to 10 items, and each item is rated on a two- to six-point scale. The two-factor structure was demonstrated in the general population: the physical health summary (PCS) and mental health summary (MCS) components could explain 60% of the total variance of the SF-36 scale scores and can be interpreted as outcome measures in clinical trials [16, 25]. We used the SF-36 scores of the survey to 2410 adults of the Chinese (HK) general population [9] as normal scores. Our study demonstrated that self-report SF-36 factors were significantly abnormal in AIP patients. The PF score and MH were lower than normal (85.74 versus 91.83, *P* = 0.001; 65.19 versus 72.79, *P* = 0.049, Table 6), while other subscales had no significant difference. The MCS and the related subscales had no significant difference with general population. We also performed comparison with other data collected from a cross-sectional survey of 21,948 cases in China from December 2005 to January 2007 of nine provinces of mainland to study the association between BMI and HRQoL in Chinese adults [12]. The GH scores were lower than normal BMI group (51.67 versus 64.7, *P* = 0.015, Table 6). Age, gender, social class gradient, and so forth had effect on the SF-36 scores [9]; most of AIP patients were young women (age: 21–47 years), and their BMI were normal or underweight, so the difference may be related to the age, sexuality, BMI, and area between general populations with AIP patients, and the difference differs from general population we choose (Table 6); it needs larger scale; the scores of the total population and the scores for groups differ in age and sexuality.

The patients who worked had lower RP scores than those who stayed at home but not across all 4 subscales. Patients without acute attack in the last year had significantly higher PF and BP scores than those with acute attack, and 1 subscale trended toward significance (GH with *P* = 0.05) and a higher PCS composite score (*P* = 0.001). 2 of 10 had received induced menopause therapy so they avoided acute attack in the last year. It revealed that reducing the attack episode might reduce the physical effects on AIP patients.

Posttraumatic stress disorder was measured by the Impact of Event Scale-Revised questionnaire. The mean IES-R score was 36.7 ± 11.8 points (26 with norm-based scoring, *P* < 0.001), and the average subscores were as follows: intrusion, 17.93 ± 5.78; avoidance, 18.48 ± 6.51; and hyperarousal, 13.26 ± 5.10.

The acute attacks of AIP had periodic occurrence without any signs and always occurred during menstruation [2, 5, 26]. The patients were afraid of attack and even nervous before every period. The prevalence of significant PTSD-related symptoms (IES-R ≥ 26) was reported [11, 20, 21, 27]. In our study, the AIP patients reported increased prevalence of PTSD-related symptoms. The patients without acute attack, at least in the last year, had better IES-R in intrusion score than those who had acute attacks. College education, marital status, and work status were not significantly different across the composite or any of the subscales. The acute attack might influence the PTSD-related symptoms of AIP patients for at least one year.

We contacted the patients via WeChat, because they could easily get the answer link and the detailed explanation about the study via WeChat, with the link including self-report questionnaires. So we got a high response rate (27/28). WeChat was a very famous and extensively used app. We could use it to follow the patients and do the questionnaire survey. It may be widely used to do the questionnaire research in the near future for it is convenient and could get the high response rate.

The prompt and accurate diagnosis of acute porphyria in China is limited by poor awareness among physicians. Measurements of urinary PBG, ALA, and porphyrins, plasma and fecal porphyrins, and erythrocyte PBG deaminase are important for the diagnosis and typing of porphyria, but in mainland of China almost none of the hospitals carry out these tests, so we use the genetic testing as the diagnostic test.

Our study has several limitations. Because AIP is a rare disease, this study was a single-center research and we only enrolled 27 cases. The results might be different from the large multicenter research. In mainland of China, hematin is not available to treat acute porphyria. 4/27 of our patients had accepted hematin treatment and could not use hematin in every attack, so we could not evaluate the relationship of hematin use with the quality of life or the PTSD symptoms.

We hope this research will help understand the impact of AIP on the quality of life of the patients from the north of China, and the new treatments that can reduce the acute attack times may help improve the quality of life.

## 6. Conclusion

The main findings of our study were that AIP patients showed significant differences in the quality of life by SF-36 questionnaire with respect to general population. They had an impaired quality of life, particularly in physical domain. Reducing the acute attack episode might help improve the PCS score. The acute attacks coursed the PTSD-related symptoms. Clinicians should pay more attention to the mental health status and impaired quality of life of the AIP patients.

## Figures and Tables

**Table 1:** Demographic characteristics of AIP patients.

Age, yrs	
18–39	25 (92.6)
40–50	2 (7.4)
Female	27 (100)
Race (Han)	27 (100)
Education	
Postcollege	6 (22.2)
College graduate	7 (25.9)
High school graduate/GED	4 (14.8)
Junior high school graduate	8 (29.6)
primary school education	2 (7.4)
Marital status	
Married	19 (70.4)
Divorced or separated	2 (7.4)
Never married	6 (22.2)
Employment	
Full-time	13 (48.1)
Homemaker	4 (14.8)
Unemployed	12 (44.4)
Age at diagnosis, yrs	
18–29	23 (85.2)
30–35	4 (14.8)
Acute attacks in the last year	
0	10
1–5	15
10	2
Without the symptoms between the attacks	18
With symptoms between the attacks	9

Values are *n* (%).

**Table 2:** Clinical manifestations.

Clinical manifestations (*n* = 27)	
Cyclical attacks	27 (100.0)
Abdominal pain	23 (85.2)
Constipation	21 (77.8)
Nausea	19 (70.4)
Paresis	13 (48.1)
Tachycardia	11 (40.7)
Confusion	8 (29.6)
Convulsion	8 (29.6)
Complaints in intermission	9 (33.3)

Values are *n* (%).

**Table 3:** PCS subscale scores and composite scores with variables.

	PF score	*P* value	RP score	*P* value	BP score	*P* value	GH score	*P* value	PCS composite	*P* value
Education										
<College graduate	88.12	0.21	76.56	0.81	75.56	0.71	58.69	0.09	43.53	0.43
College graduate	82.27		80.00		80.00		41.45		46.85	
Marital status										
Married	85.53	0.89	73.68	0.23	83.05	0.07	52.42	0.82	41.57	0.17
Single	86.25		43.75		65.89		49.88		41.24	
Work status										
Stay at home	82.08	0.15	35.42	0.02	82.93	0.21	51.5	0.98	41.57	0.08
Work	88.67		88.33		71.75		51.8		48.64	
Acute attacks										
Without attack	92.22	0.04	77.78	0.42	90.22	0.04	65.22	0.05	53.22	0.001
With attack	82.50		58.33		71.83		44.89		40.45	

**Table 4:** MCS subscale scores and composite scores with variables.

	VT score	*P* value	SF score	*P* value	RE score	*P* value	MH score	*P* value	MCS composite	*P* value
Education										
<College graduate	61.88	0.20	88.28	0.49	64.58	0.61	70.25	0.10	45.98	0.54
College graduate	52.27		81.81		75.75		57.82		48.99	
Marital status										
Married	57.63	0.89	86.84	0.69	68.42	0.92	62.95	0.36	47.16	0.70
Single	58.75		82.61		70.83		70.50		49.21	
Work status										
Stay at home	50.83	0.08	80.20	0.08	52.22	0.17	64.33	0.84	44.39	0.20
Work	63.67		90.00		82.22		65.87		50.47	
Acute attacks										
Without attack	62.22	0.74	91.67	0.43	59.26	0.83	68.00	0.42	47.12	0.70
With attack	55.59		83.82		62.75		65.41		47.53	

**Table 5:** Predictors for worsening of PTSD (IES-R subscale scores and composite scores with variables).

Variable	IES-R	*P* value	Subscales					
			Intrusion	*P* value	Avoidance	*P* value	Hyperarousal	*P* value
Education								
<College graduate	34.88	0.42	17.19	0.43	17.69	0.46	12.25	0.22
College graduate	38.64		19.00		19.00		14.73	
Marital status								
Married	35.79	0.68	17.53	0.59	18.26	0.79	13.80	0.63
Single	36.88		18.88		19.00		12.50	
Work status								
Stay at home	38.67	0.37	18.92	0.43	19.75	0.38	13.92	0.56
Work	34.60		17.13		17.47		12.73	
Acute attacks								
No attack	30.89	0.08	14.44	0.03	16.44	0.24	11.33	0.17
Have attacks	39.17		19.53		19.71		14.29	

**Table 6:** SF-36 subscale scores and comparison with norm-based scoring.

Scale	Scores	Norm-based score (HK *n* = 2410) [9]	*P* value	Norm-based score (mainland, *n* = 11836) [12]	*P* value
PF	85.74 ± 11.67	91.83	0.01	88.8	0.18
RP	64.81 ± 57.74	82.43	0.13	77.7	0.26
Bp	77.96 ± 22.81	83.98	0.18	78.9	0.83
GH	51.67 ± 25.84	55.98	0.39	64.7	0.015
VT	57.96 ± 18.96	60.27	0.53	69.1	0.005
SF	85.65 ± 23.44	91.19	0.23	82.5	0.49
RE	69.13 ± 54.64	71.67	0.81	69.7	0.06
MH	65.19 ± 19.15	72.79	0.049	72.4	0.15
